# Incentives in Diabetic Eye Assessment by Screening (IDEAS): study protocol of a three-arm randomized controlled trial using financial incentives to increase screening uptake in London

**DOI:** 10.1186/s12886-016-0206-4

**Published:** 2016-03-18

**Authors:** Gaby Judah, Ivo Vlaev, Laura Gunn, Dominic King, Derek King, Jonathan Valabhji, Ara Darzi, Colin Bicknell

**Affiliations:** Department of Surgery and Cancer, Imperial College London, St. Mary’s Campus, Praed Street, London, W2 1NY UK; Warwick Business School, University of Warwick, Scarman Road, Coventry, CV4 7AL Coventry, UK; Department of Integrative Health Science, Stetson University, 421 North Woodland Blvd., DeLand, Florida 32723 USA; Personal Social Services Research Unit, London School of Economics & Political Science, Houghton Street, London, WC2A 2AE UK; Imperial College Healthcare NHS Trust, St. Mary’s Hospital, Praed Street, London, W2 1NY UK

**Keywords:** Financial incentives, Behavioral economics, Diabetes, Diabetic retinopathy, Screening, Behavior change

## Abstract

**Background:**

Diabetes is an increasing public health problem in the UK and globally. Diabetic retinopathy is a microvascular complication of diabetes, and is one of the leading causes of blindness in the UK working age population. The diabetic eye screening programme in England aims to invite all people with diabetes aged 12 or over for retinal photography to screen for the presence of diabetic retinopathy. However, attendance rates are only 81 %, leaving many people at risk of preventable sight loss.

**Methods:**

This is a three arm randomized controlled trial to investigate the impact of different types of financial incentives (based on principles from behavioral economics) on increasing attendance at diabetic eye screening appointments in London. Eligible participants will be aged 16 or over, and are those who have been invited to screening appointments annually, but who have not attended, or telephoned to rearrange an appointment, within the last 24 months.

Eligible participants will be randomized to one of three conditions:

1. Control condition (usual invitation letter)
2. Fixed incentive condition (usual invitation letter, including a voucher for £10 if they attend their appointment)
3. Probabilistic incentive condition (invitation letter, including a voucher for a 1 in 100 chance of winning £1000 if they attend their appointment).

Participants will be sent invitation letters, and the primary outcome will be whether or not they attend their appointment. One thousand participants will be included in total, randomized with a ratio of 1.4:1:1. In order to test whether the incentive scheme has a differential impact on patients from different demographic or socio-economic groups, information will be recorded on age, gender, distance from screening center, socio-economic status and length of time since they were last screened. A cost-effectiveness analysis will also be performed.

**Discussion:**

This study will be the first trial of financial incentives for improving uptake of diabetic eye screening. If effective, the intervention may suggest a cost-effective way to increase screening rates, thus reducing unnecessary blindness.

**Trial registration:**

ISRCTN14896403, 25 February 2016

## Background

Diabetes is an increasing public health concern worldwide, with an estimated 380 million adults with diabetes globally [1]. This corresponds to 8.3 % of the world’s adult population, and is expected to rise to over 10 % by 2035. Recent data suggests that over 4 million people in the UK now have diabetes [2].

All patients with diabetes are at risk of developing diabetic retinopathy. This condition is caused by damage to small blood vessels at the back of the eyes, which reduces the blood supply. This stimulates the growth of fragile, new blood vessels in the eye, which may bleed and damage the retina, leading to sight loss. It is estimated that in England every year 4,200 people are at risk of blindness caused by diabetic retinopathy and there are 1,280 new cases of blindness caused by diabetic retinopathy [3]. It is one of the leading causes of sight loss in the UK in the working population [4] and therefore there is a significant social and financial burden associated with the condition. However, timely diagnosis and treatment can dramatically reduce the risk of blindness.

In England, all people aged 12 years and older with type 1 or type 2 diabetes are offered diabetic retinopathy screening at least annually by the Diabetic Eye Screening Programme (DES). The test involves taking a photograph of the retina, which occurs without contact with the eye. The rate of uptake for screening is 81 % [5], with a range from 7.4 % to 91.8 % across different Primary Care Trusts (PCTs) [6] (excluding the five PCTs with the highest and lowest percentages, the range is 57.7 % to 87.0 %). Therefore, a number of people with diabetes are still not being screened. This puts them at risk of developing avoidable sight loss, and is also a waste of resources in the National Health Service (NHS), due to missed appointments. Although the DES has been an unqualified success, the effectiveness of any program depends on its uptake.

Screening uptake varies geographically, and is often poor in socially deprived areas, which can exacerbate existing inequalities in health outcomes. For example, in the UK, diabetes prevalence was seen to increase with increasing deprivation, while the probability of attending diabetic retinopathy screening decreases and the prevalence of sight-threatening diabetic retinopathy among screened patients increases [7]. Since the effectiveness of any screening program is linked to the uptake by the population (and in particular uptake by those most at risk), simple, cost-effective strategies are required to realize the financial and social benefits of available sight-saving interventions, and to do this in an equitable way.

Increasing screening uptake is a challenge that can be addressed by considering other behavioral challenges in health. Reducing morbidity and mortality in the future is likely to depend as much on motivating changes in behavior as on developing new treatments or technologies, and many countries and health systems are now directing resources to this end [8, 9]. Consistent with this emphasis on preventative healthcare, screening programs currently exist in a number of other clinical areas, such as breast cancer, cervical cancer and abdominal aortic aneurysm, however, uptake can be poor. The ultimate success of a high-quality screening program depends on the uptake rate within the target population and novel solutions are required to meet the challenge of achieving this.

Recent interest has been shown in using financial incentives such as cash or vouchers, to promote desirable health behaviors (e.g. exercise, healthy eating) and discourage unhealthy ones (e.g. smoking, poor diet), and in using principles from behavioral economics to design public health interventions [10–12]. Success has been seen previously where insights from behavioral economics have been applied – namely in the areas of medication adherence and encouraging weight loss [13, 14], as there is good evidence that our response to incentive schemes can be shaped by predictable mental shortcuts. Even small incentives can positively influence choices [15], and intelligent design of incentive schemes using principles from behavioral economics will be a key strategy to cost-effectively reduce the economic and social costs of unhealthy behavior [16].

Financial incentives have been seen to be more effective in increasing performance of infrequent behaviors (e.g. vaccinations) rather than in more sustained behaviors (e.g. smoking) [15, 17]. As screening usually requires discrete one-off behaviors, incentives may be particularly effective in increasing their uptake. Other examples include programs to offer vouchers to young women who attend cervical smear testing [18]. Offering vouchers can reduce loss-to-follow-up in women who have had an abnormal cervical smear test. Since financial based incentives for healthy behavior are already being used, for example by large employers or health insurance providers [19–22], we require evidence that they work, and if they do, which method is most cost effective. This will avoid wasting public resources.

While some feel that financial incentives are controversial as they may be a form of coercion, appropriately targeted incentives can reduce inequalities in health outcomes [12, 15, 23]. Furthermore, as people often do not act in line with their intentions [24], financial incentives have been proposed as a way to help people’s actions align more closely with their true preferences, and therefore to enhance as opposed to restrict autonomy [25]. A survey of patients with diabetes found that while some had strong concerns about incentives, many people supported the principle of incentivizing good behaviors, and more people were more in favor of vouchers, or small cash payments as opposed to larger payments [26]. Support for incentives was more common in those aged between 40 and 64, and in more deprived groups. A recent review of studies on acceptability of financial incentives for health behaviors found that incentives tend to be acceptable if they are effective and cost-effective, and if they benefit recipients and wider society [27].

This study will investigate the applicability of behavioral economics and financial incentives to increasing diabetic retinopathy screening uptake in non-attenders, by testing the impact and cost-effectiveness of two different incentive schemes. This randomized controlled trial takes place in London, which has both high and low levels of deprivation and specific populations with poor attendance. This study would represent one of the first robust investigations of the use of incentives in diabetic eye screening, and of incentives specifically inspired by behavioral economics [28, 29]. The results will be of particular interest to policy makers running screening programs and seeking cost-effective ways to increase uptake, but could have a wider impact amongst those looking to change behavior in other health domains.

### Research questions

In this study we will compare two different incentive schemes for diabetic retinopathy screening, with a usual-care control group, to determine:

1. Are incentives an effective strategy to encourage participation in the screening programme?
  While screening for diabetic retinopathy is an effective strategy for reducing blindness, to be cost-effective the screening programme requires good attendance. However, attendance rates could be improved. This study will investigate whether targeted financial incentives can increase screening participation.
2. Does the design of the financial incentive scheme affect its effectiveness in influencing participation in health screening?
  There are many ways in which incentives to encourage screening participation could be delivered. Two different types of financial incentive will be compared to see which is most effective: a fixed £10 incentive, or a prize draw with a 1 in 100 chance of winning £1000.
3. Does the choice of incentive scheme, if successful, attract patients who have a different demographic or socioeconomic status to those who attend screening regularly?
  A particular concern is that those in deprived socio-economic groups are less likely to attend screening [7, 30], exacerbating existing inequalities in health. By investigating the impact of our incentive schemes on the demographic profile of those who attend, we hope to learn more about the way in which incentives might be developed to target specific health inequalities. We will obtain information about age, gender, postcode and hence social deprivation status, and distance from screening center.
4. Is offering incentives a cost-effective strategy for enhancing participation?
  In the current financial environment it is also important to ensure that any interventions are cost-effective. Economic evaluation using well-established economic models will be performed to determine value for money.

## Methods

### Study population and eligibility

Eligible participants will be identified from the Diabetic Eye Screening Programme (DES) prior to commencement of the study by 1st Retinal Screen Ltd (who at the time of the study were providing the DES service in the Kensington, Chelsea and Westminster Clinical Commissioning Groups in West London). Participants will be patients aged sixteen years and older, who have been invited to screening in the last 24 months on a yearly basis, but who have failed to attend or to have contacted the screening service to rearrange an appointment. A minimum two month period will be left between any of the standard invitation letters and enrolment into the trial to ensure that patients are not enrolled who are late to contact the screening service but who still intend to do so. Due to contractual requirements, the normal, annual invite process will continue for trial participants.

### Study design and procedure

The study design will be a three-arm randomized controlled trial. The effect of two different types of financial incentives will be compared to a control group with no incentives. The list of eligible participants identified by 1st Retinal Screen Ltd will be randomized by the statistician (according to an anonymous identifier), prior to the start of the study. No personal information about participants will be shared by 1st Retinal Screen Ltd. For *n* eligible participants provided in the list, they will be indexed according to numbers generated at random with double precision, to avoid duplicates. Participants will then be sorted from smallest to largest according to this random index. Within the sorted list and using the 1.4:1:1 randomisation ratio, we will assign: (1) the lowest n/3.4 participants to the fixed incentive group; (2) the following n/3.4 participants to the probabilistic incentive group; and (3) the remainder of the participants to the control group. We will round n/3.4 to the closest integer, if needed. Participants will be sent a letter by 1st Retinal Screen Ltd inviting them to their appointment, however they will be able to reschedule this appointment once (by phoning a number given on the letter), and still be eligible for the incentive. The letter sent will be determined by the intervention condition to which participants are assigned; these are described below.

**Fig. 1:**
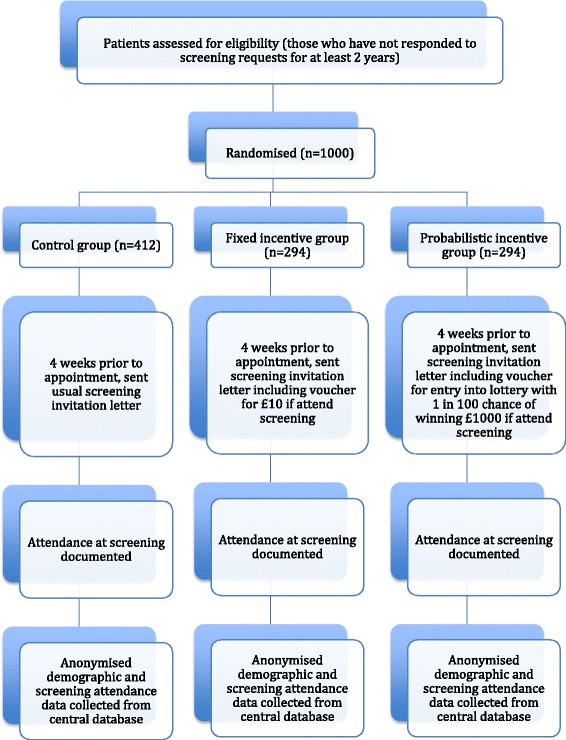
Flow diagram of study procedure. There will be oversampling at the randomisation stage, to allow for the sample size to be reached even if patients become ineligible between randomisation and the invitation letter being sent

The study will take place at a DES clinic based within St Mary’s Hospital, Imperial College Healthcare NHS Trust, in London, UK. Dedicated clinics will be arranged for trial participants, and for each of the intervention groups, so that non-trial patients are not aware of incentives offered to trial patients, and so participants within different groups are not aware of the different incentive conditions. A researcher will be present at the screening sessions for the intervention groups, in order to be able to answer any questions about the incentive, and to give the cash incentive to those in the fixed incentive group. As there will be designated clinics for each intervention group, it will not be possible for the researcher or screener to be blinded to the group assignment. However, as attendance at the clinic is the primary outcome, the study results cannot be biased by the lack of blinding at this stage. The study procedure is shown in Fig. 1.

### Conditions

The design of the intervention conditions took into account established psychological phenomenon from Prospect Theory (for which Daniel Kahneman was awarded the 2002 Nobel prize in economics [28]). In particular, the two intervention conditions were based on the insights of ‘reference points’ and ‘overweighting of small probabilities’. The incentive vouchers will also contain expiry dates of the date of the screening, to introduce some aspect of ‘loss aversion’, whereby losing a reward is more powerful than gaining a reward [31].

Both incentive letters were shown to patients within Westminster Diabetes User Group, and they were deemed to be acceptable. There was also a patient representative within the trial management team, who assisted with and approved the letter design.

#### Control group

Participants in this group will receive the standard invitation letter from the Screening Service, which invites patients to a fixed appointment at a particular date and time (there is a number to call if patients need to rearrange their appointment). The letter will be sent four weeks before the appointment date.

#### Fixed incentive group

**Fig. 2:**
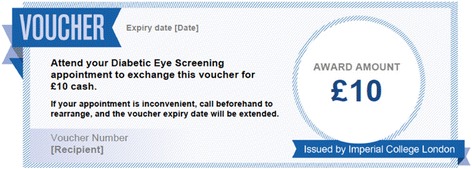
Image of voucher added to the invitation letter for the fixed incentive group. The following text was also added to the standard letter: We know that some patients invited for diabetic eye screening do not attend their appointment. Imperial College London is looking to see whether financial incentives help people to attend, and this work is being conducted through our clinics. Once you have been screened, you can exchange this voucher for £10 cash. Please bring this letter with you when you attend your screening appointment. If you have any questions about the financial incentive, please email (address)

Participants in this group receive the standard invitation letter as in the control group, however this will include additional text and a voucher offering a financial incentive of £10 after screening is completed. The voucher is shown in Fig. 2.

This amount of £10 was chosen due to the idea of reference points, which can be illustrated by the finding that offering a very small incentive is effective at encouraging people to pick up their HIV test result, but increasing the value of the incentive has little effect [32]. Therefore, the intervention will offer an incentive of £10 (equivalent to minimal hourly wage for UK workers >21), to cover the opportunity cost to the patient in terms of their time or travel costs.

#### Probabilistic incentive group

Participants in this group will receive the standard invitation letter, including additional text and a voucher offering entry into a prize draw for a 1 in 100 chance of winning £1000 following attendance at screening.

This condition was designed with an understanding of the phenomena that people overvalue small probabilities, which explains the popularity of lotteries and insurance [28]. It has been demonstrated that given fixed resources in an incentive programme, it can be more effective to offer lotteries, compared to smaller individual rewards [33]. We will offer a lottery where the expected value matches the incentive level offered in the other study condition. It may be that overweighting of small probabilities will make a lottery offering e.g., '1 % chance for £1000' more attractive than £10 for certain, so the former may be a more effective incentive for participation in screening.

To inform the intervention design, a survey was conducted of over fifty patients who attended the vascular and diabetic foot clinics at Imperial College Healthcare NHS Trust. Each was asked which of the following incentives was most attractive for them personally:

- £5 for sure
- A 1 in 10 chance of winning £50
- A 1 in 100 chance of winning £500
- A 1 in 1000 chance of winning £5000

All patients either answered (a) or (d). There was no one who thought that the answers (b) or (c) were more attractive. This suggests that patients fall into two groups – ‘risk avoiders’ and ‘risk seekers’, as defined relatively to the available set of choice options. (See [34], for discussion of evidence for such relativity of risk preferences.) We have chosen incentives with lottery odds 1 in 100 (1 %) chance of winning a monetary prize, and not smaller probability (1 in 1000), because people have cognitive difficulties in comprehending, and dealing with, very small probabilities (see [28]). Therefore, because it is difficult to conceptualise what 1 in 1000 chance means, individuals may ignore the odds and focus only on the prize, which may induce further nonlinearities in behavior in addition to probability overweighting. The magnitude of the offered prize in the probabilistic incentive group (£1000) was selected so that it is multiple of the £10 payoff offered in the other intervention condition: if there is a 100 % chance to get £10 in the fixed incentive group (justified on the grounds of real time and travel costs), then it makes fair sense to offer £1000 when the probability is hundred times smaller (i.e. the ‘expected value’ of the incentive is the same in both conditions). This amount is also the highest lottery incentive that could be offered based on the number of people in the trial.

**Fig. 3:**
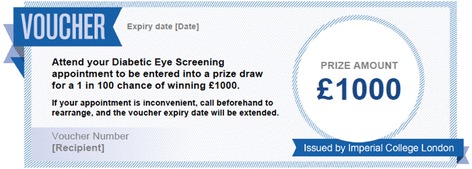
Image of voucher added to the invitation letter for the probabilistic incentive group. The following text was also added to the standard letter: We know that some patients invited for diabetic eye screening do not attend their appointment. Imperial College London is looking to see whether financial incentives help people to attend, and this work is being conducted through our clinics. Once you have been screened, you will be entered into a prize draw where you will have a 1 in 100 chance of winning £1000. Please bring this letter with you when you attend your screening appointment. If you have any questions about the financial incentive, please email (address)

The prize draw will be conducted using a computer program giving each participant an exact 1 in 100 probability of winning. The probabilistic incentive voucher is shown in Fig. 3. To ensure that a prize is awarded, as required for legal reasons, if none of the participants who attend win the prize following the individual draws, then one participant will be chosen at random as the winner, from all participants who attended from the probabilistic incentive group. When a winner of the probabilistic incentive is identified, their contact details will be given to the researcher by 1st Retinal Screen Ltd. and they will be sent a letter to arrange payment of the £1000 prize.

### Measures

The primary endpoint is the proportion of invitees who attend screening in each incentive group. This anonymised information will be extracted from the Screening Service database. For those participants who attend their screening appointment, data on their screening outcome score will be collected, aggregated by intervention group.

Demographic information will be collected for all invited participants on age, deprivation score and distance from screening center. Age data will given in deciles, starting from the lowest eligible age of 16 (i.e. 16–25, 26–35 etc.) with those aged 86 years and over being in the highest category. Deprivation scores will be measured based on the Indices of Multiple Deprivation score [35], which is based on postcodes, and will use the address to which the invitation is posted. This data will be given in deciles to ensure participant confidentiality. Distance to screening site will be measured based on the straight-line distance between the address on the day of screening, and the screening site, rounded to the nearest kilometre. Baseline demographics will also be collected for: gender, years registered, and years since last attended screening.

If participants telephone the screening center to opt not to participate in screening, the reason(s) provided will be recorded.

In all groups, when patients arrive, the screener will ask them for any reasons why they have not attended their past few appointments, in order to see if there are differences between intervention groups, and to explore common barriers to attendance in this hard-to-reach group. Reasons given will be noted in a table containing common reasons for non-attendance, along with the intervention group assignment. The reasons suggested are those given by patients in some informal telephone research conducted by 1st Retinal Screen Ltd (shown in Table 1).

**Table 1:** Suggested reasons for past non-attendance

Suggested reasons for non-attendance	
Forgot	
Didn't know had an appointment	
Didn't get round to coming	
Was out of the country	
Started experiencing problem with eyes	
Did not feel obviously had problems with eyes	
Couldn't get time off work	
Didn't understand why needed to be screened	
Thought optician did it	
Family commitments	
Too ill to attend	
Seen privately	
Problems with transport	
Do not consider themselves diabetic	
Under the care of Hospital Eye Services	
Other - please specify	

Following completion of the study, the dataset will be generated by the data manager at 1st Retinal Screen Ltd, given a database search of their system to extract all relevant attendance and demographic data.

### Sample size calculation

The primary endpoint is the proportion of the study group who attend their diabetic retinopathy screening appointment. As the study group will comprise patients who have not attended screening for at least two years, including some who have never attended, despite previous interventions to encourage them to attend, this is a very hard to reach population. Attendance in the control group is expected to be extremely low, such as a nominal 1 %. As the eligible study population also tends to be the sickest, with the greatest risk of having retinopathy, and of losing their sight, even a small increase in attendance would have clinical and social benefit.

An increase in attendance of 10 % was deemed clinically significant. A 10 % increase was also considered achievable, as a study found that smoking cessation rates among employees of a large company increased from 5 % to 14.7 % with financial incentives [36], and in another study, warfarin adherence among subjects requiring anticoagulation management improved from 65 % to 97.8 % with a lottery based financial incentive [14]. With two intervention groups, each being compared to the control group, maximum statistical efficiency is achieved by randomising 1.4:1:1. Combining this with an assumed increase in attendance of 10 %, from 1 % in the control arm to 11 % in an intervention arm, there would be 95 % power if 1000 participants were recruited in total (412 in the control group, and 294 in each of the intervention groups). This sample size would also give the study at least 85 % power to detect a smaller increase from 1 % to 7.5 %, which would also be a clinically significant improvement in attendance rates. Should attendance in the control group turn out to be 5 %, the study would still have approximately 85 % power to detect an increase in attendance of 10 % to a level of 15 % in an intervention group [37].

Data from 1st Retinal Screen Ltd suggests that the study group could comprise over 1000 patients. This would allow oversampling at the randomisation stage, so that the intended sample size will still be reached even if participants become ineligible following randomisation (the most common reason for this is most likely to be due to participants attending their routine screening appointments).

### Statistical analysis

#### Summary statistics

The primary outcome will be the count and proportion of attenders (i.e. attendance rate) by treatment group and demographics.

Secondary data will be the sight outcome scores from the screening test, aggregated by treatment group. This can be classified as disease compared to no disease, or sight outcome (annual recall, digital surveillance, refer to ophthalmology, refer to ophthalmology-urgent).

Those who become ineligible after randomisation and before receiving the invitation letter will be excluded from the analysis due to no longer meeting study inclusion criteria. However, a distribution of number becoming ineligible by treatment group, as well as reasons for ineligibility will be provided. Some participants may become ineligible after receiving the invitation letter, yet it can be assumed that the letter itself does not cause ineligibility, and so would occur at random across the experimental conditions. Therefore participants becoming ineligible after receiving the invitation letter will be excluded from the analysis.

The count and proportion of participants opting not to participate, or rearranging appointments, will be presented by reasons provided, as well as according to treatment group and demographics. If participants rearrange more than one appointment, they become ineligible for the incentive, and would be counted as a non-attender (even those in the control group). The count and proportion of participants who were classified as non-attenders for this reason will be provided, and they will also be included in a sensitivity analysis for comparison, where they will be classified as attenders.

#### Pairwise comparisons

After summary statistics are analyzed, pairwise comparisons of success, or attendance, rates (absolute risk differences) will be performed between the following groups using Chi-square tests adjusting for multiple comparisons:

- Control vs. Fixed incentive
- Control vs. Lottery incentive
- Fixed vs. Lottery incentive

Risk differences and risk ratios (i.e., relative risks), along with their 95 % confidence intervals, will be presented to assess whether any significant differences between groups exist. This analysis will address the first two research questions of whether financial incentives, and then the design of the incentive scheme have an impact on screening attendance.

Further analyses will be conducted to explore the third research question about whether the incentive schemes attract patients with a different socioeconomic or demographic status. Although groups will be checked after randomisation on the basis of demographic factors, an exploratory subgroup analysis will be performed for adjustment of treatment effect by accounting for the available demographic covariates listed above using a multivariate logistic regression analysis, and performing model selection using a backward stepwise removal process based on a 0.05 significance level; covariate-adjusted differences in success, or attendance, rates between treatment groups will be computed. Significant associations will be identified by those with *p*-values < 0.05, while possible trends toward significance will be identified from 0.05 ≤ *p*-values < 0.10. With anticipated relatively low success rates globally, resulting estimates from these subgroup analyses could be unstable [38, 39], hence affecting possible reliability of the estimates which is why we emphasize the *exploratory* subgroup analysis.

To answer the third research question, comparisons will also be made to those who are classified as regular ‘current’ attenders (i.e., those who have attended at least 2 appointments in the past 3 years) to assess possible differences through demographic covariates and secondary outcome data between regular attenders and non-attenders.

#### Cost-effectiveness analysis

In order to investigate the fourth research question about the cost-effectiveness of the interventions, a health economics analysis will be conducted. The trial results will provide data on the differential rate of attendance to screening for each group. To assess the short-term cost-effectiveness of the incentives, the cost per additional screening attendee will be calculated as the cost of screening divided by the observed increase in the number of screening attendees for each pairwise comparison of groups.

A longer-term, 5 year, perspective of the cost-effectiveness of the incentives will be assessed using a Markov model. The model will assess the cost-effectiveness from the NHS perspective using the differential rates of screening attendance for each group. The long-term effects of attending screening in terms of the sensitivity of screening, treatment costs and quality of life, as well as the long-term effects of not attending screening, will be determined from the published literature. The costs across the groups will differ only in the monetary incentives provided.

The analysis pertaining to the third research question will indicate if the incentives for attending screening are associated with individual characteristics. If this is the case, the published literature will be consulted to determine if the significant characteristic(s) also impact on the costs and/or quality of life of individuals with diabetes. If so, this will be reflected in the modelling of future costs and quality of life. Sensitivity analysis will be conducted to determine the impact upon cost-effectiveness of changes in the key parameters within the model.

### Ethical approval

The sponsor for the study is Imperial College London. The study has been reviewed by the London Riverside National Health Service Research Ethics Committee, from which it received a favourable opinion (reference number 14/LO/1779).

### Consent

Informed consent will not be obtained from research participants. This is similar to other screening trials, as it is not possible to gain informed consent before the screening invitations are sent out. Furthermore, if patients were aware of what is being investigated in the trial, it would jeopardise the reliability of the findings. The London Riverside National Health Service Research Ethics Committee waived the need for study participants to provide informed consent as part of the approval process. However, following the conclusion of the trial, patients will be informed by letter that they were included in a trial, and informed of the results.

### Trial management

The study has been funded by the National Institute for Health Research (NIHR), Health Services and Delivery Research Programme, project number 12/64/112. The research was supported by the NIHR Biomedical Research Centre based at Imperial College Healthcare NHS Trust and Imperial College London. The study was registered with ISRCTN on 25 February 2016, with number 14896403. The protocol is version 1.5, dated 4 August 2015. The trial sponsor assisted with obtaining ethical approvals for the study, will assist with any protocol modifications, and conduct any auditing or monitoring of the trial here necessary.

The conduct of the trial will be managed by the trial management team (TMT) comprising individuals from a broad range of areas of expertise relevant to the study. The TMT will meet regularly to discuss trial progress, deal with any reported adverse events, and produce outputs for scientific and public dissemination given the results of the study. A subset of the TMT with expertise in data analysis will agree any changes to the analysis plan, and monitor the data management. TMT members from 1st Retinal Screen Ltd, Imperial College London and the statistician will have access to the dataset. 

## Discussion

This study is a novel, randomized controlled trial to investigate the impact of two different types of financial incentives on increasing attendance at diabetic retinopathy screening. The design of the incentives has been based on established principles from behavioral economics [40]. This intervention therefore has the potential to reduce unnecessary sight loss, and to generate large savings in public funds.

Based on the ‘Future Sight Loss’ report [41] and data from the NHS Diabetic Eye Screening website [42], we estimate potential societal savings in the UK of £1.6 million if the £10 is successful in increasing take-up of screening by 10 %. From these reports, it was estimated that 0.94 % of patients are at risk of blindness due to diabetic retinopathy, and the rate of blindness amongst those at risk is 30.5 %. Therefore, if 10 % of the estimated 448,000 individuals with diabetes who fail to attend screening annually were to attend due to the incentive, this would correspond to detection of 423 people who would have been at risk of blindness, and prevention of 129 cases of blindness. Given the annual, per person cost of blindness and sight loss is £12,466 the potential economic savings of reducing screening non-attendance is £1,608,114. The cost of screening the 1,000 study subjects is estimated to be £20,700. The cost of providing a £10 incentive to the total sample will be £10,000. Thus a rough estimate of the overall potential saving, if the study is successful in improving screening, is £1,577,414.

As there is evidence of public support for incentive schemes that are effective and cost-effective [27], if this intervention has the expected impact, it may be an acceptable way to prevent unnecessary blindness due to diabetic retinopathy.

## Abbreviations

DES: Diabetic Eye Screening
ISRCTN: International Standard Randomised Controlled Trial Number
NHS: National Health Service
NIHR: National Institute for Health Research
PCT: Primary Care Trust
TMT: Trial management team
UK: United Kingdom
